# Job demand and control in mid-life and physical and mental functioning in early old age: do childhood factors explain these associations in a British birth cohort?

**DOI:** 10.1136/bmjopen-2014-005578

**Published:** 2014-10-15

**Authors:** Mikaela B von Bonsdorff, Rachel Cooper, Diana Kuh

**Affiliations:** 1 Department of Health Sciences, Gerontology Research Center and University of Jyväskylä, Jyväskylä, Finland; 2 MRC Unit for Lifelong Health and Ageing at UCL, London, UK

**Keywords:** EPIDEMIOLOGY, OCCUPATIONAL & INDUSTRIAL MEDICINE, PUBLIC HEALTH, SPORTS MEDICINE

## Abstract

**Objectives:**

Adverse work-related exposures have been linked with decreased physical and mental functioning in later life, however, whether childhood factors explain the associations between work exposures and functioning is unknown. Our aim was to investigate if job demand and control in mid-life were related to self-reported physical and mental functioning in early old age and whether childhood factors explained these associations.

**Design:**

Prospective cohort study.

**Setting:**

England, Scotland and Wales.

**Participants and outcome measures:**

Data come from the UK Medical Research Council National Survey of Health and Development, a cohort with follow-up since birth in 1946. 1485 occupationally active study members had data available on job demand and control in mid-life and on physical and mental functioning assessed using the Short Form-36 questionnaire at 60–64 years.

**Results:**

Those with higher job control in mid-life had better physical functioning than those who reported lower job control (β 0.51, 95% CI 0.02 to 1.01, p=0.04 adjusted for adult confounders). Those with higher job demand in mid-life had poorer mental functioning (β −0.82, 95% CI −1.14 to −0.51, p<0.001). Associations between job control and mental functioning were similar but less pronounced. Adjustment for childhood factors (father's and mother's educational attainment, parents’ interest in school at age 7 and cognitive ability at age 8) partially explained the association between job control and physical functioning, but did not explain the association between job demand and mental functioning.

**Conclusions:**

Job demand and control in mid-life are differentially associated with mental and physical functioning in early old age and some of these associations may be partially explained by childhood factors.

**Strengths and limitations of this study**

- Using data from a nationally representative British birth cohort followed up across life to early old age, we were able to use prospectively collected data on job demand and control in mid-life and self-reported mental and physical functioning in early old age.
- With the availability of a wealth of prospectively collected data on factors in childhood we were able to explore the influence of these on the main associations of interest which few other studies are able to do.
- We used the validated and widely used Short Form-36 physical and mental functioning component scores to ascertain physical and mental functioning.
- Job demand and control were not ascertained using a standard questionnaire as these data were collected in 1982 and 1989 when the use of standardised questionnaires was not common. Attrition in prospective studies with follow-up across the whole of life is to be expected. Although this might introduce bias, the cohort have been found at age 53 to be representative in most respects of the national population born at a similar time and the distribution of occupational class and unemployment at ages 60–64 was similar to the 2001 England Census reference population.

## Introduction

In the occupationally active work force, adverse work-related exposures such as high-job demand, low-job control or a combination of the two, that is, work-related mental strain, have been linked with an increased prevalence of chronic conditions such as cardiovascular disease, diabetes, depression and obesity,^1–4^ increased use of hospital care services^5^ and premature mortality.^6^ ^7^ Some evidence is available on the short-term negative associations between job strain in mid-life and physical and mental functioning,^8^ ^9^ but so far, only a few studies have explicitly investigated the long-term consequences of job strain for physical and mental functioning in older age. One study showed that higher job strain was associated with decreased self-reported physical and mental health functioning after retirement^10^ and another one that job-related stress was associated with disability in old age.^11^ This may be due to physiological responses of the activation of the hypothalamic-pituitary-adrenal axis controlling stress-responses which in the case of prolonged stress such as job strain may impact several body systems consequently increasing the risk of poor physical and mental functioning.^12^ The long-term effects of adverse job exposures on physical and mental functioning in later life are of particular interest in light of current increases in the demands of working life, coupled with expectations that ageing employees will transition into retirement at older ages.^13^

Childhood factors may influence the risk of adverse work-related exposures in adulthood^14^ and help to explain the associations between these adverse work-related exposures and later health outcomes. There are a number of proposed pathways by which childhood factors could be associated with workplace exposures and functioning in early old age. First, there is evidence of the continuity of socioeconomic position across life,^15^ that is, individuals from lower socioeconomic backgrounds are less likely to attain higher levels of education and thus are more likely to end up in professions with lower job control.^14^ Lower socioeconomic position across the life course has been shown to predict poorer physical functioning in mid-life^16^ Second, individuals who have been subjected to social adversity in childhood might be more vulnerable to higher job demands than those with no such experiences.^17^ Third, persons with lower childhood cognitive ability may be more susceptible to the negative impacts that work-related exposures have on functioning because of lower resilience to cope with this stress.^18^ A few studies have investigated the role of childhood factors in the relationship between job strain and health outcomes such as cardiovascular disease and its risk factors. In the 1958 British birth cohort, factors such as cognitive ability in childhood and adolescence were found to explain a significant proportion of the positive association between job control and levels of glycosylated haemoglobin.^19^ However, in the Whitehall II study, childhood factors such as father's education and number of siblings explained only a modest proportion of the relationship between psychosocial factors at work and coronary heart disease.^20^ To the best of our knowledge, there have been no studies investigating whether early life factors explain the relationships between adverse work-related exposures and physical and mental functioning, outcomes of particular relevance for ageing societies. Using prospective data spanning six decades from the nationally representative Medical Research Council (MRC) National Survey of Health and Development (NSHD), the aims of this study were to: (1) investigate if job demand and control in mid-life were related to self-reported physical and mental functioning in early old age; and (2) examine whether parental characteristics and childhood cognitive ability explained these associations.

## Methods

### Participants

The Medical Research Council National Survey of Health and Development (MRC NSHD) is a socially stratified sample of 5362 singleton births to married women in March 1946 in England, Scotland and Wales that has been followed from birth every 2 years in childhood and adolescence and in adulthood at the ages of 26, 31, 36, 43, 53 and 60–64 years.^21^ ^22^ At the latest follow-up at 60–64 years, the study team was still in contact with 3163 members of the original cohort. Of those not contacted, 718 had died, 594 had previously withdrawn from the study, 567 lived abroad and 320 had been untraceable for more than 10 years. Of the 3163, 2661 (84.1%) were successfully contacted^22^ and completed a postal questionnaire and/or clinic or home visit. Postal questionnaires which included the Short Form-36 (SF-36) on physical and mental functioning were completed by 2293 members of the cohort.

### Physical and mental functioning in early old age

Self-reported physical and mental functioning was ascertained using the SF-36 health survey questionnaire at ages 60–64.^23^ The questionnaire consists of 36 items which generate eight subscales including physical functioning, role limitations due to poor physical health, bodily pain, general health perception, vitality, social functioning, role limitations due to poor emotional health and mental health. Physical and mental component summaries were calculated using all eight subscales with different sex-specific weights which were obtained from a population sample in the UK. The scores were transformed into T-scores so that the overall mean for the participants was 50 (SD 10), with a higher score indicating better functioning.^24^ If less than half of the items in each of the subscales were missing, standard procedures were applied to impute values.^25^

### Job demand and control in midlife

Data on employment and work characteristics of study members were collected during home visits by research nurses on several occasions during the work career.^26^ We drew on Karasek's job demand and control model^27^ when constructing the job demand and control variables. At age 36 (in 1982), occupationally active study members were asked three questions describing their job demand: (1) if they often have deadlines to meet at work (yes/no) and, if so, how they find it (very stressful=0, fairly stressful=1 or not particularly stressful=2); (2) if emotionally work takes: a lot=0, a moderate amount=1 or very little=2 of them; and (3) if at the end of an average day at work they were feeling often pressed for time (yes=1 or no=0). The first question was combined into 1=no deadlines or deadlines not particularly stressful, 2=has deadlines and finds it fairly stressful and 3=has deadlines and finds it very stressful. After recoding the questions so that a higher value indicated higher demand, we calculated a summary score of the three items (Cronbach α 0.55) describing job demand ranging from 0 (lowest) to 5 (highest job demand) (see online supplementary table S1).

At age 43 (in 1989), occupationally active study members were asked two questions describing job control: (1) if they have the possibility to learn new things through their work (often=2, sometimes=1 or seldom/never=0) and (2) if they have a good deal of say in how they do their work (often=2, sometimes=1 or seldom/never=0). We calculated a summary score of the two items (Cronbach α 0.50) describing job control ranging from 0 (lowest) to 4 (highest job control) (see online supplementary table S1).

### Covariates

The theoretical framework which we had developed and used to guide our selection of covariates was partially based on findings from earlier literature.^18^ ^25^ ^28^ ^29^

#### Childhood factors

Childhood factors which have been shown to be associated with other measures of physical and mental functioning in mid-life and early old age in the NSHD and which were, on the basis of evidence from other studies,^14^ also expected to be associated with job characteristics were identified a priori as being potentially important and were included in analyses. *Mother's and father's education* were reported when study members were aged 4 and were coded into primary, secondary or higher. The *level of interest (low, average, high) that the parents had in the progress of the study members at school* at age 7 was assessed by school teachers on the basis of parents’ contact with the school. *Childhood cognition* was assessed at age eight with four tests on reading comprehension, pronunciation, vocabulary and nonverbal reasoning, devised by the National Foundation for Educational Research. These raw scores were standardised and then summed and transformed into a standardised z score indicating general cognitive ability in childhood.

#### Potential adult confounders

Occupational class was ascertained at age 43 and the British Registrar General's classification was used to assign study members into a manual or non-manual occupational class. Smoking history ascertained at ages 60–64 was coded as current, former or never smoker. Study members were asked about participating in exercise, vigorous activity or sports at ages 60–64, which was coded as inactive (reported no participation), participating in relevant activities one to four times per month or participating at least five times per month. Weight and height, measured at ages 60–64 were used to calculate body mass index, (BMI) (kg/(m^2^)).

### Statistical analyses

Using linear regression models we investigated the associations between job demand and job control and physical and mental functioning adjusting for gender. To test for potential gender differences, we analysed the interaction ‘gender*job demand/job control’ on physical and mental functioning, but because these terms were not statistically significant (all p values >0.064), subsequent analyses were pooled by gender, with gender-specific sensitivity analyses also performed. We then made adjustment for the other work exposure (job demand/control), followed by childhood factors (mother's and father's education, parents’ interest in study member's school at age 7 and cognitive ability at age 8), then adult confounders (occupational class at age 43 and BMI, smoking history and level of physical activity at age 60–64) and finally for all covariates simultaneously. In subsequent analyses, the contribution of childhood factors to the associations between job demand/control and physical and mental functioning were investigated by first adjusting the model separately for each, and then simultaneously adjusting for all childhood factors. The percentage change in the unstandardised regression coefficients was calculated using the standard formula of (β base model—β adjusted model)/(β base model)×100%.

Of the 5362 original members of the cohort, 3322 (62%) participated in the 1982 follow-up at age 36, of them 2482 (74.7%) were occupationally active and had answered the questions on job demand and of them, 1601 (64.5%) had completed the SF-36 at ages 60–64. Of the 3262 (60.8%) study members participating in the 1989 follow-up at age 43, 2835 (86.9%) had answered the questions on job control and 1971 (69.5%) had completed the SF-36 at ages 60–64. Altogether 1485 study members had data available on all the main variables used in this study; job demand at age 36, job control at age 43 and the SF-36 at ages 60–64. In order to obtain a with complete data on all the main variables and covariates, we imputed values for covariates where data were missing using multiple imputation (mother's education n=145, father's education n=151, parental interest in progress of study member at school n=171, cognitive ability at age 8 n=144, occupational class n=4 and BMI n=216, physical activity n=232 and smoking n=40). A total of 20 imputed data sets were created using all variables in the analyses together with data on the same covariates collected at other close data sweeps. Regression models were first performed using complete data available for all main variables and covariates and then using the multiply imputed data sets, with the effect estimates combined using Rubin's rules. While the results were largely the same, we present findings on imputed data; the models using complete data are presented in online supplementary table S2. All tests were performed two tailed, the level of significance was set at p<0.05 and analyses were carried out using SPSS IBM V.20.0 (SPSS, Armonk, New York, USA, IBM Corp).

## Results

Of the participants who had been occupationally active in mid-life, approximately 61% were men, 69% belonged to a non-manual occupational class and approximately 70% had a mother or father with primary education only. At ages 60–64, about 10% of the participants were current smokers and 52% were physically inactive (table 1).

**Table 1:** Characteristics of National Survey of Health and Development (NSHD) cohort members who were occupationally active in mid-life and whose physical and mental functioning had been assessed at ages 60–64, proportions and percentages (n, %) unless stated otherwise

	Sample	All		Men n=901		Women n=584	
Work exposures							
Job demand at age 36*, mean SD	1485	1.53	1.53	1.65	1.41	1.36	1.29
Job control at age 43*, mean SD	1485	2.92	1.07	3.06	1.02	2.71	1.12
Adult characteristics							
Occupational class at age 43	1481						
Manual		464	31.2	275	30.7	189	32.4
Non-manual		1017	68.5	622	69.3	395	67.6
Body mass index at ages 60–64, mean SD	1269	27.82	4.05	27.83	4.05	27.80	5.18
Smoking history at ages 60–64	1445						
Current		153	10.3	87	10.0	66	11.6
Ex-smoker		829	55.8	525	60.0	304	53.2
Never smoked		463	31.2	262	30.0	201	35.2
Physical activity level at ages 60–64	1253						
Inactive		765	51.5	475	62.9	290	58.2
1–4 times per month		190	12.8	108	14.3	82	16.5
5 times or more per month		298	20.1	172	22.8	126	25.3
Childhood exposures							
Mother's education, primary	1340	1050	70.7	626	76.9	424	80.6
Father's education, primary	1334	970	65.3	577	71.6	393	74.4
Cognitive ability at age 8†, mean SD	1341	0.16	0.81	0.12	0.81	0.21	0.80
Parental interest in study member's school at age 7	1314						
Low		92	6.2	61	7.7	31	6.0
Intermediate		586	39.5	359	45.3	227	43.6
High		636	42.8	373	47.0	263	50.5

*Formation of the work exposure variables is explained in online supplementary table S1.

†Standardised z-score across the original NSHD cohort.

In unadjusted analyses there were graded associations between job demand/control in mid-life and physical and mental functioning in older age such that those with higher job demand scores had lower mean mental functioning scores and those with higher job control scores had higher mean physical functioning scores (table 2). Physical and mental functioning scores are presented according to individual questions on job demand and control in online supplementary table S1.

**Table 2:** Mean levels of self-reported physical and mental functioning in early old age according to work exposures in mid-life

			Physical component*			Mental component*		
	n	(%)	Mean	SD	p Value†	Mean	SD	p Value†
Job demand at age 36‡								
0	424	28.6	51.4	10.5	0.82	52.9	7.3	0.005
1	394	26.5	50.9	10.2		52.1	8.0	
2	309	20.8	51.6	9.7		51.7	8.0	
3	209	14.1	50.9	9.4		50.9	9.8	
4	107	7.2	50.4	10.3		50.1	8.1	
5	42	2.8	52.2	8.1		50.7	8.6	
Job control at age 43‡								
0	49	3.3	49.1	10.5	0.044	50.0	7.9	0.27
1	116	7.8	50.0	11.3		51.0	8.5	
2	276	18.6	50.2	11.0		51.8	8.7	
3	506	34.1	51.4	9.6		52.0	8.4	
4	538	36.2	52.0	9.4		52.2	7.5	

*A higher score indicates better physical and mental functioning.

†Analyses of variance.

‡A higher score indicates higher job demand/control, formation of work exposure variables explained in online supplementary table S1.

Coefficients from linear regression models confirmed that participants who reported higher job control in mid-life had better physical functioning at ages 60–64 than those who reported lower job control, β (difference in mean physical functioning score per 1 unit increase in job control) 0.51, 95% CI 0.02 to 1.01, p=0.04 adjusted for gender, job demand and adult confounders (table 3). Job demand in mid-life was not associated with physical functioning in analyses adjusted only for gender; however, adjustment for adult confounders strengthened the association so that it reached statistical significance, suggesting negative confounding.

**Table 3:** Unstandardised regression coefficients (β) and 95% CIs for physical and mental functioning in early old age according to work exposure in mid-life n=1485

	Model 1		Model 2		Model 3		Model 4		Model 5	
	β (95% CI)*	p Value	β (95% CI)*	p Value	β (95% CI)*	p Value	β (95% CI)*	p Value	β (95% CI)*	p Value
Physical functioning										
Job control	0.70 (0.22 to 1.18)	0.004	0.76 (0.27 to 1.26)	0.002	0.58 (0.08 to 1.09)	0.024	0.51 (0.02 to 1.01)	0.040	0.50 (−0.006 to 1.00)	0.053
Job demand	−0.10 (−0.47 to 0.28)	0.60	−0.23 (−0.61 to 0.15)	0.24	−0.36 (−0.75 to 0.03)	0.072	−0.48 (−0.86 to −0.10)	0.013	−0.49 (−0.87 to −0.11)	0.012
Mental functioning										
Job control	0.42 (0.03 to 0.81)	0.035	0.62 (0.23 to 1.02)	0.002	0.52 (0.11 to 0.93)	0.014	0.47 (0.06 to 0.88)	0.025	0.42 (0.001 to 0.84)	0.050
Job demand	−0.62 (−0.92 to −0.31)	<0.001	−0.72 (−1.03 to −0.41)	<0.001	−0.76 (−1.08 to −0.45)	<0.001	−0.82 (−1.14 to −0.51)	<0.001	−0.83 (−1.15 to −0.51)	<0.001

Model 1 adjusted for gender; model 2 adjusted for gender and the other work exposure; model 3 same adjustments as model 2 plus father's and mother's education, parents interest in study member's school at age 7 and cognitive ability at age 8; model 4 same adjustments as model 2 plus occupational status at age 43 and smoking, physical activity and BMI at ages 60–64; model 5 adjusted for all covariates included in models 2, 3 and 4.

*Differences in mean physical and mental functioning scores per 1 unit increase in job demand/control score, formation of work exposure variables explained in online supplementary table S1.

BMI, body mass index.

Higher job control was associated with better mental functioning at ages 60–64, but adjustment for childhood factors and adult confounders attenuated this association (as indicated by the reduction in the size of effect estimates when comparing models 3 to 5 with model 2 in table 3). Those participants who had higher job demand in mid-life had poorer mental functioning in early old age compared to those with lower job demand, β (difference in mean mental functioning score per 1 unit increase in job demand) −0.82, 95% CI −1.14 to −0.51, p<0.001 adjusted for gender, job control and adult confounders (table 3). Adjustment for childhood factors had little impact.

When investigating the influence of each childhood factor on these associations separately, individual adjustments for father's and mother's educational attainment, parents’ interest in study member's school and childhood cognitive ability reduced the size of the positive association between job control and physical functioning between 5.3 and 14.5%; mutual adjustment for all childhood factors was estimated to explain 23.7% of this association (see online supplementary table S3). The positive association between job control and mental functioning was less pronounced and adjusting for childhood factors attenuated the association less. The childhood factors included did not explain the negative association found between job demand and mental functioning at ages 60–64.

## Discussion

In participants of a British birth cohort study who had been occupationally active in mid-life, there was some evidence to suggest that lower job demand was associated with better mental functioning and higher job control with better physical and mental functioning decades later, around the age of retirement. Childhood factors, namely cognitive ability, parental education and parental interest in study member's schooling, appeared to partially explain the positive association between job control in mid-life and physical functioning in early old age. The association between higher job demand and lower mental functioning was robust; adjustment for selected childhood factors and potential adult confounders had very little influence on the strength of this association.

Our findings on the associations between job demand and control and self-reported physical and mental functioning in early old age are in line with the few published studies showing that work-related stress and job strain in mid-life have negative short-term^8^ ^9^ and long-term effects^10^ ^11^ on physical and mental functioning. Similar to our present findings, the associations with job demand were found to be more pronounced for mental than physical functioning in the French Gazel cohort.^10^ Earlier findings from the NSHD and the Helsinki Birth Cohort Study have shown that various negative familial factors in childhood are related to decreased objectively measured^18^ ^28^ ^29^ and self-reported^25^ ^30^ physical and mental functioning in mid-life and early old age. To the best our knowledge, this is the first study to investigate prospectively the contribution of childhood factors in the relationships of job demand and control with self-reported physical and mental functioning in early old age. The findings in the few studies investigating the role of early life factors in the relationship between job strain and cardiovascular disease have been inconsistent. A non-significant effect of pre-employment factors, defined as family history of coronary heart disease, father's education and socioeconomic position, number of siblings and adult height, was reported for the longitudinal association between job strain and coronary heart disease in the Whitehall II study.^20^ Conversely, in the 1958 British birth cohort early life exposures were found to explain up to 50% of the associations between different workplace exposures such as low job control and increased levels of risk factors for cardiovascular disease.^19^ The inconsistent findings may be related to differences in the characteristics of the study populations, in the childhood factors available and in the frequency of assessments of the work characteristics.

As discussed in the introduction, some of the mechanisms that are hypothesised to underlie the associations between job strain and physical and mental functioning in older age relate to the biological, psychological and social responses to chronic stress. Employees working in high-strain jobs have been shown to be more likely to suffer from the negative physiological and psychological effects of excessive stress such as cardiovascular and metabolic dysregulation and depression,^12^ ^31^ making those people exposed to high-job strain more vulnerable to the ageing process, which could be manifested as lower physical and mental functioning in later life.^32^ It has also been hypothesised that exposure to adverse psychological and social factors in childhood may predispose an individual to the negative effects of job strain.^17^ ^33^ This could result in those people who have experienced early life adversities coping differently with job strain and being more frequently negatively affected by this than individuals who did not experience early life adversity. It has been shown that childhood environmental factors, such as parental education, familial situation and parents’ level of interest in offspring schooling as well as the child's own cognitive ability may influence responses to later adverse work-related exposures.^14^ Childhood cognitive ability, although being socially graded, has been shown to be related to both work characteristics^34^ and functioning in adulthood^18^ above and beyond its relationship with socioeconomic position. In our study, allowing for childhood factors attenuated the association between job control and physical functioning, supporting this observation.

Another possible explanation of our findings is that individuals working in high-strain jobs are more likely to report unhealthy behaviours such as smoking and physical inactivity^35^ which are established risk factors for lower levels of physical and mental functioning.^36^ ^37^ Studies have also found that early life adversities might set an individual on an unfavourable behavioural trajectory that extends across life into older age.^38^ Our findings suggest that the relationships between job control and self-reported mental and physical functioning may be partly explained by unhealthy behaviours and poorer socioeconomic circumstances in adulthood, however, this was not the case for job demand and mental functioning.

Another possibility that we need to consider is that our findings, in particular those on mental functioning, may be partially explained by reverse causality; pre-existing personality traits and poor mental health might detrimentally influence both the perception of work exposures^39^ and mental functioning in later life and thus lead to strengthening of the observed relationship between adverse work exposures in mid-life and later mental functioning.

In the present study higher job demand was associated with poor mental functioning in later life, and this was not explained by any of the childhood or adult lifestyle factors we adjusted for. Conversely, the association between perceived job control and mental functioning was partially explained by these factors. A potential explanation of this difference is that we were not able to take account of the genetic and biological factors related to stress responses^12^ which have been shown to vary according to childhood factors^40^ that may be more likely to underlie the associations between job demand and mental functioning. These underlying pathways need to be further explored in other studies with the relevant data.

The study had several strengths. First, data came from the nationally representative MRC NSHD, a well-characterised cohort including persons with a wide range of professions. Second, we were able to use prospective data collected across life and there are a wealth of data on childhood exposures that we selected into the analyses a priori based on earlier findings. Third, we used the validated and widely used SF-36 physical and mental functioning component scores^23^ to ascertain physical and mental functioning. Despite these strengths some limitations also need to be acknowledged. First, our measures of job demand and control were only ascertained once during working life, the two measures were collected 7 years apart and neither were ascertained using standard questionnaires. However, at the times of data collection in 1982 and 1989, when details on work were collected, the use of standardised questionnaires was not common. The cohort was stable in terms of occupational class during the two measurement points as 57% belonged to the non-manual and 26% to the manual class at both assessments and only 17% crossed this divide. To explore the validity of our job demand and control variables, we compared the scores with other work-related data in NSHD in mid-life. For example, the participants were asked at age 43 if they often had a backlog of work to get through. Those who answered ‘often’ had a mean job demand score of 2.00 (SD 1.4) at age 36 and those who answered ‘seldom/never’ a score of 1.06 (SD 1.1) (analysis of variance (ANOVA) p<0.001). At age 43, the participants were asked how often they wished they were doing a different job. Those who answered ‘often’ had a mean job control score of 2.0 (SD 1.2) and those who answered ‘seldom/never’ a score of 3.2 (0.9) (ANOVA p<0.001). Given there are correlations between the two variables used in the main analyses, that is, job demand and control, and other work-related characteristics we cannot fully rule out the possibility that the main associations found may be partially explained by other work-related characteristics. Second, attrition, which is inevitable in such a long prospective study, might cause bias, although the MRC NSHD has been found at age 53 to be representative in most respects of the national population born around that time^41^ and the occupational class and unemployment profile of the MRC NSHD at ages 60–64 to be similar to the 2001 England Census reference population.^22^ In the NSHD at ages 36 and 43, 2140 occupationally active study members had data available on job demand and control. Of these occupationally active study members, those who had data on physical and mental functioning at ages 60–64 (n=1485) had more often a father with secondary education (χ^2^ test p=0.002) and were working in a non-manual profession at age 43 (χ^2^ test p<0.001), compared to those who did not have data on functioning (n=655).

In conclusion, evidence suggests that lower job demand in mid-life may be associated with better mental functioning and higher job control with better physical functioning around the age of retirement. Some of these associations were partly explained by childhood factors such as parental education and interest in study member's school and childhood cognitive ability as well as adult lifestyle factors. Although, the influence of these factors was modest, these findings highlight the complex associations acting across life that are likely to underlie the relationships between adverse job exposure and later health outcomes which is often not possible to examine in part due to limited availability of data from earlier life in studies with detailed occupational measures. The present findings indicate that higher job demand in particular may have adverse long-term psychological effects on individuals around the age of retirement, which may in turn decrease the chances of healthy ageing.
